# Supplementary material for: Predicting visual function by interpreting a neuronal wiring diagram
Source: Nature. 2024 Oct 2;634(8032):113–23. doi: 10.1038/s41586-024-07953-5 (PMC11446822; doi:10.1038/s41586-024-07953-5)
Supplement: Supplementary file 6 — CRF and ERF predictions for individual TmY4 and TmY9 cells. Analogous to Supplementary Data 3, but for TmY target types. Shown are the top four monosynaptic pathways, the strongest pathway passing through each of the top ten intermediary types (ranking from Extended Data Fig. 7), and the trisynaptic pathway Tm1–TmY–Dm3–TmY (see the section entitled Prediction of spatial normalization). [file 41586_2024_7953_MOESM6_ESM.zip › DataS4/TmY9q/720575940615498818.pdf]

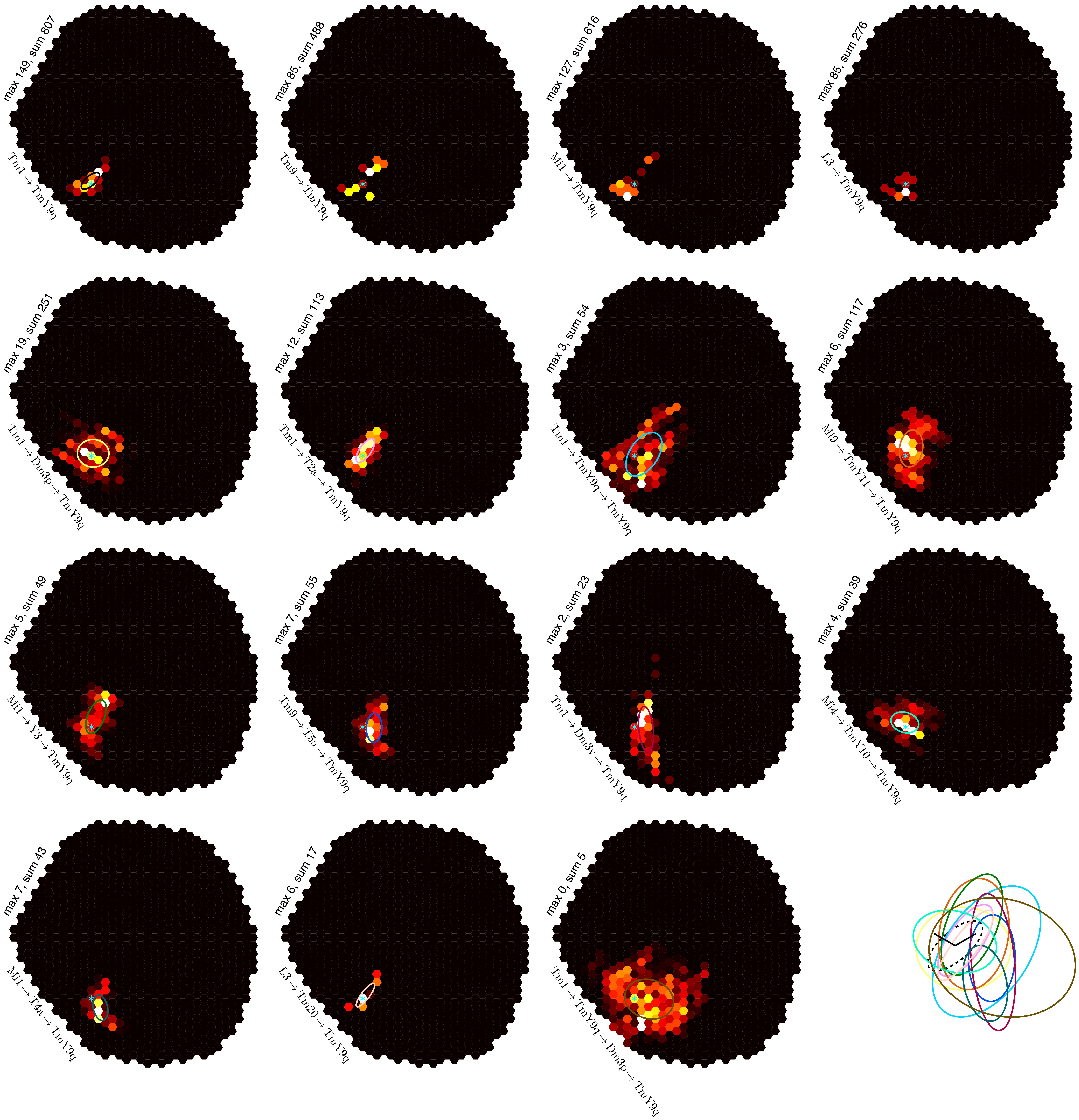
max 149, sum 807
Tm1 → TmY9q
max 85, sum 488
Tm9 → TmY9q
max 127, sum 616
Mi1 → TmY9q
max 85, sum 276
L3 → TmY9q
max 19, sum 251
Tm1 → Dm3p → TmY9q
max 12, sum 113
Tm1 → T2a → TmY9q
max 3, sum 54
Tm1 → TmY9q → TmY9q
max 6, sum 117
Mi9 → TmY11 → TmY9q
max 5, sum 49
Mi1 → Y3 → TmY9q
max 7, sum 55
Tm9 → T5a → TmY9q
max 2, sum 23
Tm1 → Dm3v → TmY9q
max 4, sum 39
Mi4 → TmY10 → TmY9q
max 7, sum 43
Mi1 → T4a → TmY9q
max 6, sum 17
L3 → Tm20 → TmY9q
max 0, sum 5
Tm1 → TmY9q → Dm3p → TmY9q
